# Robustness of superconductivity to external pressure in high-entropy-alloy-type metal telluride AgInSnPbBiTe_5_

**DOI:** 10.1038/s41598-022-11862-w

**Published:** 2022-05-12

**Authors:** Md. Riad Kasem, Yuki Nakahira, Hitoshi Yamaoka, Ryo Matsumoto, Aichi Yamashita, Hirofumi Ishii, Nozomu Hiraoka, Yoshihiko Takano, Yosuke Goto, Yoshikazu Mizuguchi

**Affiliations:** 1 Department of Physics, Tokyo Metropolitan University, 1-1 Minami-Osawa, Hachioji, 192-0397 Japan; 2 RIKEN SPring-8 Center, Sayo, Hyogo 679-5148 Japan; 3 International Center for Young Scientists (ICYS), National Institute for Materials Science, Tsukuba, Ibaraki 305-0047 Japan; 4 International Center for Materials Nanoarchitectonics (MANA), National Institute for Materials Science, Tsukuba, Ibaraki 305-0047 Japan; 5 National Synchrotron Radiation Research Center, Hsinchu, 30076 Taiwan

**Keywords:** Phase transitions and critical phenomena, Superconducting properties and materials

## Abstract

High-entropy-alloy (HEA) superconductors are a new class of disordered superconductors. However, commonality of superconducting characteristics of HEA materials is unclear. Here, we have investigated the crystal and electronic structure, and the robustness of superconducting states in a HEA-type metal telluride (*M*Te; *M* = Ag, In, Sn, Pb, Bi) under high pressure, and the results were compared with the pressure effects for a middle-entropy system (AgPbBiTe_3_) and a reference system of PbTe. When the crystal structure is CsCl-type, all phases show superconductivity under high pressure but exhibit different pressure dependences of the transition temperature (*T*_c_). For PbTe, its *T*_c_ decreases with pressure. In contrast, the *T*_c_ of HEA-type AgInSnPbBiTe_5_ is almost independent of pressure, for pressures ranging from 13.0 to 35.1 GPa. Those results suggest that the robustness of superconductivity to external pressure is linked to the configurational entropy of mixing at the *M* site in *M*Te. Since the trend is quite similar to previous work on a HEA (Ti–Zr–Hf–Nb–Ta), where the robustness of superconductivity was observed up to ~ 200 GPa, we propose that the robustness of superconductivity under high pressure would be a universal feature in HEA-type superconductors.

## Introduction

The superconductivity of disordered materials has been extensively studied because of an observation made on the insulator-superconductor transition or disorder-induced superconductivity^1–3^. In addition, recent studies on BiS_2_-based layered superconductors have shown the importance of controlling the local structural disorder to improve superconducting properties^4–7^. As a new category of disordered superconductors, high-entropy alloys (HEAs), which are alloys containing five or more elements with an atomic concentration between 5 and 35%^8,9^, have been extensively studied, leading to the discovery of a wide range of HEA superconductors^10–12^. One of the notable features is the difference in characteristics of superconductivity among conventional metal or alloy superconductors, superconducting thin films, and HEA superconductors^11,13^. Namely, the dependence of *T*_c_ on valence electron count (VEC) for HEAs exhibits interesting trends when that was compared with those for classical alloys (metals) and amorphous alloys^13^. In the CsCl-type HEAs, the *T*_c_-VEC exhibits opposite trend to that for the classical and amorphous alloys. In the α-Mn phase, although the trend of the *T*_c_-VEC exhibits commonality among HEAs, classical, and amorphous alloys, *T*_c_ for HEAs is clearly low. Therefore, detailed investigations are necessary to understand universality of local structure and superconductivity characteristics in HEA-type superconductors. Furthermore, the robustness of superconductivity to extremely high pressure (HP), for pressures up to 190 GPa, in an HEA, (TaNb)_0.67_(HfZrTi)_0.33_, was observed^14^. The high configurational entropy of mixing (Δ*S*_mix_) reduces the Gibbs’s free energy, and therefore stabilization of the phase is expected. The equation for calculating the Gibb’s free energy is Δ*G* = Δ*H* − *T*Δ*S*_mix,_ where Δ*H* is the enthalpy and *T* is the absolute temperature. Δ*S*_mix_ can be calculated using the equation Δ*S*_mix_ = − *R* ∑_*i*_* c*_*i*_ln*c*_*i*_, where, *R* is the gas constant and *c*_i_ is the atomic ratio of the element (*i*)^8^. The discovery of the robustness of superconductivity under an extremely high pressure was considered to be related to the high Δ*S*_mix_, but similar robustness of superconductivity under an extremely high pressure and a high transition temperature (*T*_c_) of 19 K was observed in a low-entropy Nb–Ti alloy^15^. Therefore, the effects of Δ*S*_mix_ on the crystal structure, electronic structure, and superconducting properties of HEAs are still unclear. To address this issue, further investigation of HP effects on HEA-type materials is needed.

We recently developed superconducting HEA-type compounds, in which one of the crystallographic sites is alloyed with the criterion similar to HEAs^16^. Since compounds with two or more crystallographic sites have unique chemical bonds, various (novel) effects of the introduction of HEA site to electronic and structural properties would be expected, which is so-called cocktail effect in the field of HEAs. In the layered BiS_2_-based superconductor *RE*(O,F)BiS_2_, the rare-earth site (*RE*) was alloyed with five different *RE* elements^17^, and the improvement of superconducting properties (bulk nature of superconductivity) with increasing Δ*S*_mix_ was observed^18^. *T*_c_ was not affected by Δ*S*_mix_, and therefore its insensitivity to Δ*S*_mix_ in the BiS_2_-based superconductor is explained using a two-dimensional structure, since similar insensitivity was also observed in an *RE*123 cuprate with an HEA-type *RE* site^19^. In contrast, *T*_c_ of NaCl-type metal telluride, which is the target phase of this study, shows that its sensitivity to Δ*S*_mix_ of HEA-type systems is larger than that of metal tellurides with one or two constituent elements at the metal site^20,21^.

On the basis of the facts described above, we considered that the metal telluride system is suitable for the discussion of the effects of high Δ*S*_mix_ on the crystal structure (phase stability) and superconducting properties under high pressure. Here, we studied the crystal structure, electronic structure, and superconducting properties of three tellurides (*M*Te; *M* = Ag, In, Sn, Pb, Bi) with different Δ*S*_mix_ values at the *M* site: PbTe (Δ*S*_mix_ = 0), AgPbBiTe_3_ (Δ*S*_mix_ = 1.1*R*), and AgInSnPbBiTe_5_ (Δ*S*_mix_ = 1.6*R*). The pressure evolutions of the crystal structure and electronic transport properties were reported in previous studies^22–26^. Although PbTe is a semiconductor under ambient and low pressures, it undergoes metallization under high pressures, and a superconducting transition is observed for pressures above 15 GPa^22,23^. PbTe undergoes structural transitions as follows: cubic NaCl-type (*Fm*–3*m*) structure under low pressures, orthorhombic (*Pnma*) structure under moderate pressures, and cubic CsCl-type (*Pm–*3*m*) structure under HP^24–26^. There is a report on synthesis and thermoelectric properties of AgPbBiTe_3_, but the structural and physical properties of AgPbBiTe_3_ have not been reported^27^. In this study, we established a phase diagram of the crystal structures, as well as one for superconductivity versus pressure for both AgPbBiTe_3_ and AgSnInPbBiTe_5_. We also studied PbTe to examine the effects of configurational entropy of mixing. The pressure phase diagrams for the three tellurides are used to compare the crystal and electronic structures in detail. The results show that the pressure phase diagram of the crystal structure is largely modified by the effect of Δ*S*_mix_. Furthermore, we found that the pressure dependence of *T*_c_ in the HP phase (CsCl-type phase) exhibits a clear difference among the three compounds. In the HP phase, the *T*_c_ for PbTe decreases with pressure, but that for HEA-type AgInSnPbBiTe_5_ exhibits a flat dependence, which suggests that the superconducting state of AgInSnPbBiTe_5_ is robust to pressure. Our present findings show an analogical conclusion in the case of (TaNb)_0.67_(HfZrTi)_0.33_^14^ and suggest that the HEA effects universally enhance the robustness of superconductivity under high pressure.

## Results

### Electrical resistance

**Figure 1:**
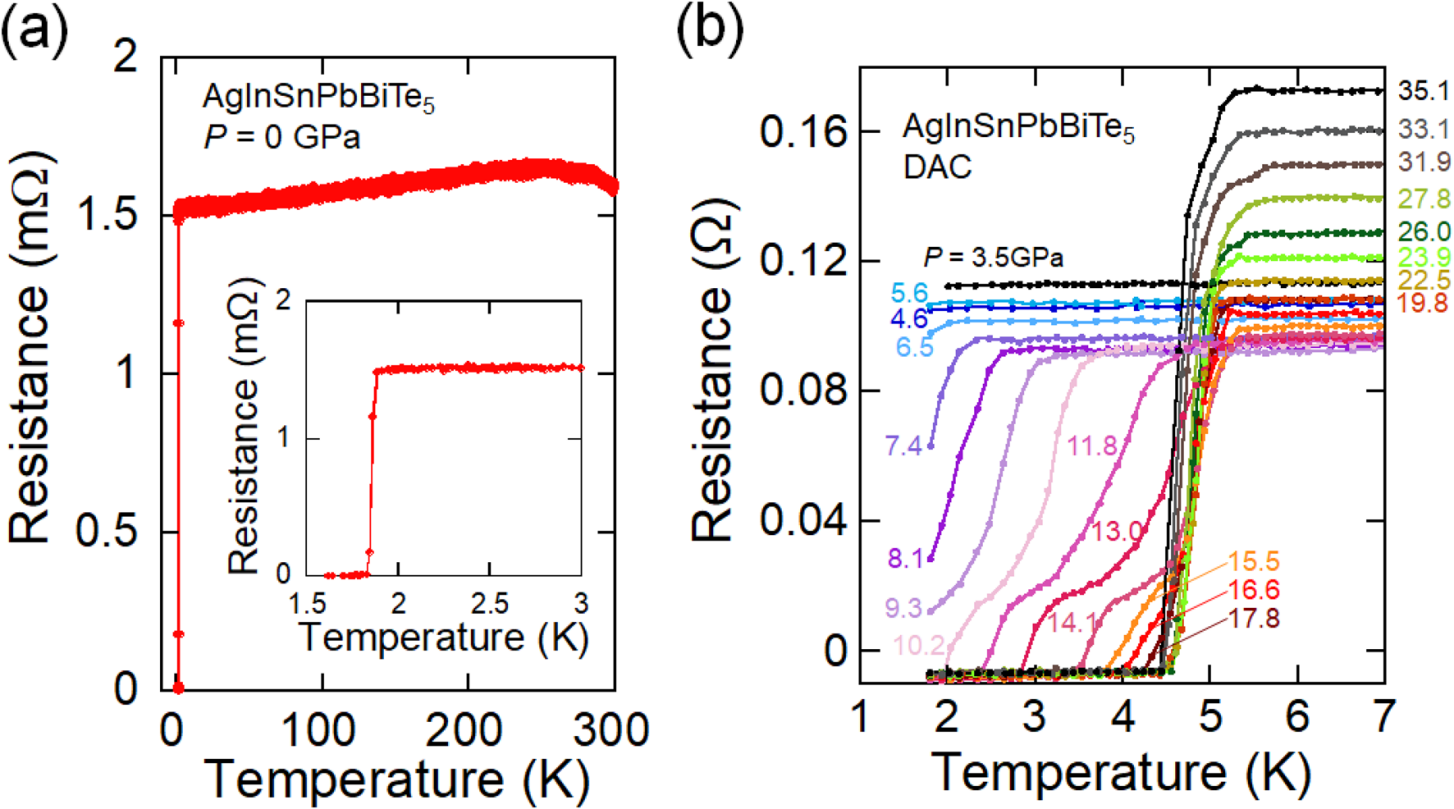
Superconducting transition in AgInSnPbBiTe_5_ under high pressure. (**a**) Temperature dependence of electrical resistance of AgInSnPbBiTe_5_ measured using the conventional four-probe method. (**b**) Temperature dependences of electrical resistance of AgInSnPbBiTe_5_ measured using a diamond anvil cell (DAC).

Figure 1a shows the temperature dependence of the electrical resistance of AgInSnPbBiTe_5_ measured using the conventional four-probe method at ambient pressure. $T_{{\text{c}}}^{{{\text{zero}}}}$ was 1.8 K, which is slightly lower than that reported in previous research^20^. We measured the electrical resistance (at National Institute for Materials Science) after several days since the sample was synthesized by HP annealing (at Tokyo Metropolitan University), and a slight aging effect of *T*_c_ was noticed in *M*Te superconductors synthesized under high pressure^28,29^. We consider that the slight decrease in *T*_c_ can be understood by the change in the internal strains because the XRD patterns do not show a remarkable change after aging in the HP-synthesized *M*Te samples. Therefore, we continued to perform resistance measurements under high pressure using a diamond-anvil cell (DAC).

**Figure 2:**
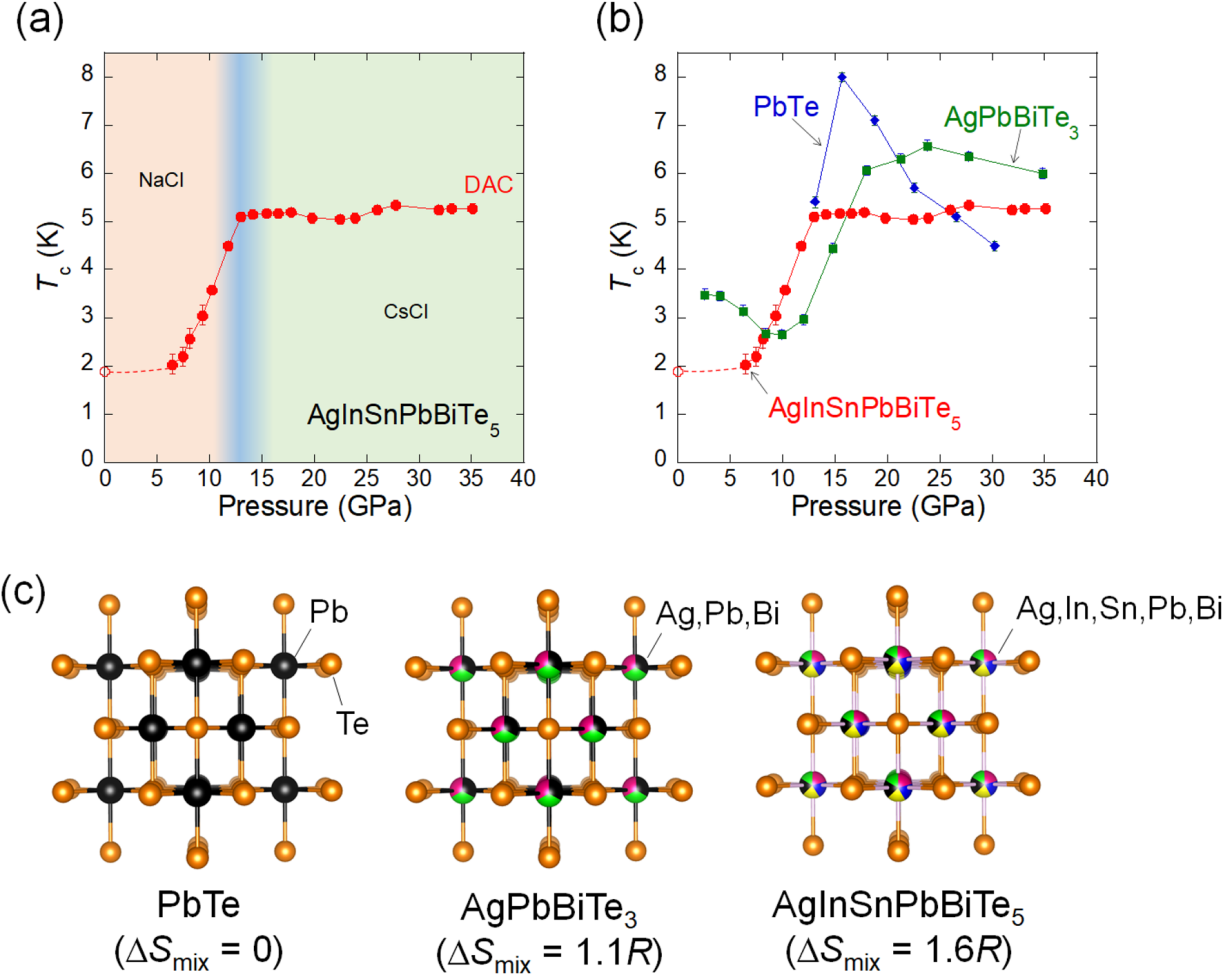
Pressure evolutions of *T*_c_ of metal tellurides with different configurational entropy of mixing. (**a**) Pressure dependence of $T_{{\text{c}}}^{{{\text{onset}}}}$ of AgInSnPbBiTe_5_. Open and filled circles indicate the data taken without a pressure cell, and the data that was measured with DAC. The structural types are indicated according to structural analyses in Fig. 3d. (**b**) Pressure dependences of $T_{{\text{c}}}^{{{\text{onset}}}}$ for PbTe, AgPbBiTe_3_, and AgInSnPbBiTe_5_. (**c**) Schematic images of NaCl-type crystal structure and configurational entropy of mixing (Δ*S*_mix_) at the *M* site of PbTe, AgPbBiTe_3_, and AgInSnPbBiTe_5_.

Figure 1b shows the temperature dependences of the electrical resistance of AgInSnPbBiTe_5_ measured under various pressures with a DAC. Normal-state resistance decreases with pressure, for pressures up to 9.3 GPa and increases with pressure for pressures above 15.5 GPa. An onset of the superconducting transition was observed at 2 K under 6.5 GPa, and the zero-resistance state was observed at $T_{{\text{c}}}^{{{\text{zero}}}}$ = 2 K under 10.2 GPa. At low pressures below 6.5 GPa, the onset of superconductivity was not observed. We consider that *T*_c_ does not remarkably change but slightly decreases with pressure because a slight decrease in $T_{{\text{c}}}^{{{\text{onset}}}}$ was observed in the NaCl-type phase of AgPbBiTe_3_. In the middle-pressure range, two-step transitions were observed, which would be due to the coexistence of two phase under relatively inhomogeneous pressures generated by DAC. As shown in Fig. 2a, the $T_{{\text{c}}}^{{{\text{onset}}}}$ monotonously increased with pressure until it reached 13.0 GPa, and then it became insensitive to pressure for pressures ranging from 13.0 to 35.1 GPa (*P* = 35.1 GPa is the chosen maximum pressure for the experiment). The results indicate that the *T*_c_ in the CsCl-type structure of AgInSnPbBiTe_5_ is independent of applied pressure_,_ while lattice constant decreases with pressure. The highest $T_{{\text{c}}}^{{{\text{zero}}}}$ and $T_{{\text{c}}}^{{{\text{onset}}}}$ observed in the experiment on AgInSnPbBiTe_5_ are 4.5 K and 5.3 K, respectively. See Fig. S1 for the determination criterion for $T_{{\text{c}}}^{{{\text{onset}}}}$.

To compare the pressure evolution of *T*_c_ in AgInSnPbBiTe_5_ with that of PbTe and AgPbBiTe_3_, the pressure dependences of $T_{{\text{c}}}^{{{\text{onset}}}}$ for PbTe and AgPbBiTe_3_ were examined (see Fig. 2c for structural difference and Figs. S2 and S3 for the resistance measurements), and the resulting $T_{{\text{c}}}^{{{\text{onset}}}}$-*P* plots are shown in Fig. 2b. PbTe is a semiconductor at ambient and low pressures, but exhibits a pressure-induced superconducting transition above 17 GPa in the CsCl-type structure^22^. At higher pressures, $T_{{\text{c}}}^{{{\text{onset}}}}$ of PbTe monotonously decreases with pressure. In the case of AgPbBiTe_3_, superconductivity was observed at *P* > 2.6 GPa, and at this instance $T_{{\text{c}}}^{{{\text{onset}}}}$ reached 6.5 K. The $T_{{\text{c}}}^{{{\text{onset}}}}$ for AgPbBiTe_3_ slightly decreases at high pressures. In AgInSnPbBiTe_5_, superconductivity is observed at low pressures as well because the low-pressure phase, having a NaCl-type structure, itself is a metal and shows superconductivity under ambient pressure (Figs. 1a, 2a). As demonstrated in the next section, the crystal-structure type under high pressure is CsCl-type for all the compounds. However, the trend of the pressure dependences of $T_{{\text{c}}}^{{{\text{onset}}}}$ in the CsCl-type structure exhibit a clear difference among PbTe, AgPbBiTe_3_, and AgInSnPbBiTe_5_. The main findings of this study are that the robustness of superconductivity to pressure in HEA-type AgInSnPbBiTe_5_ is similar to that observed in (TaNb)_0.67_(HfZrTi)_0.33_^14^. To validate the conclusion, we investigated the pressure evolutions of the crystal structure and the electronic structure for those *M*Te samples under high pressure.

### Crystal structure

**Figure 3:**
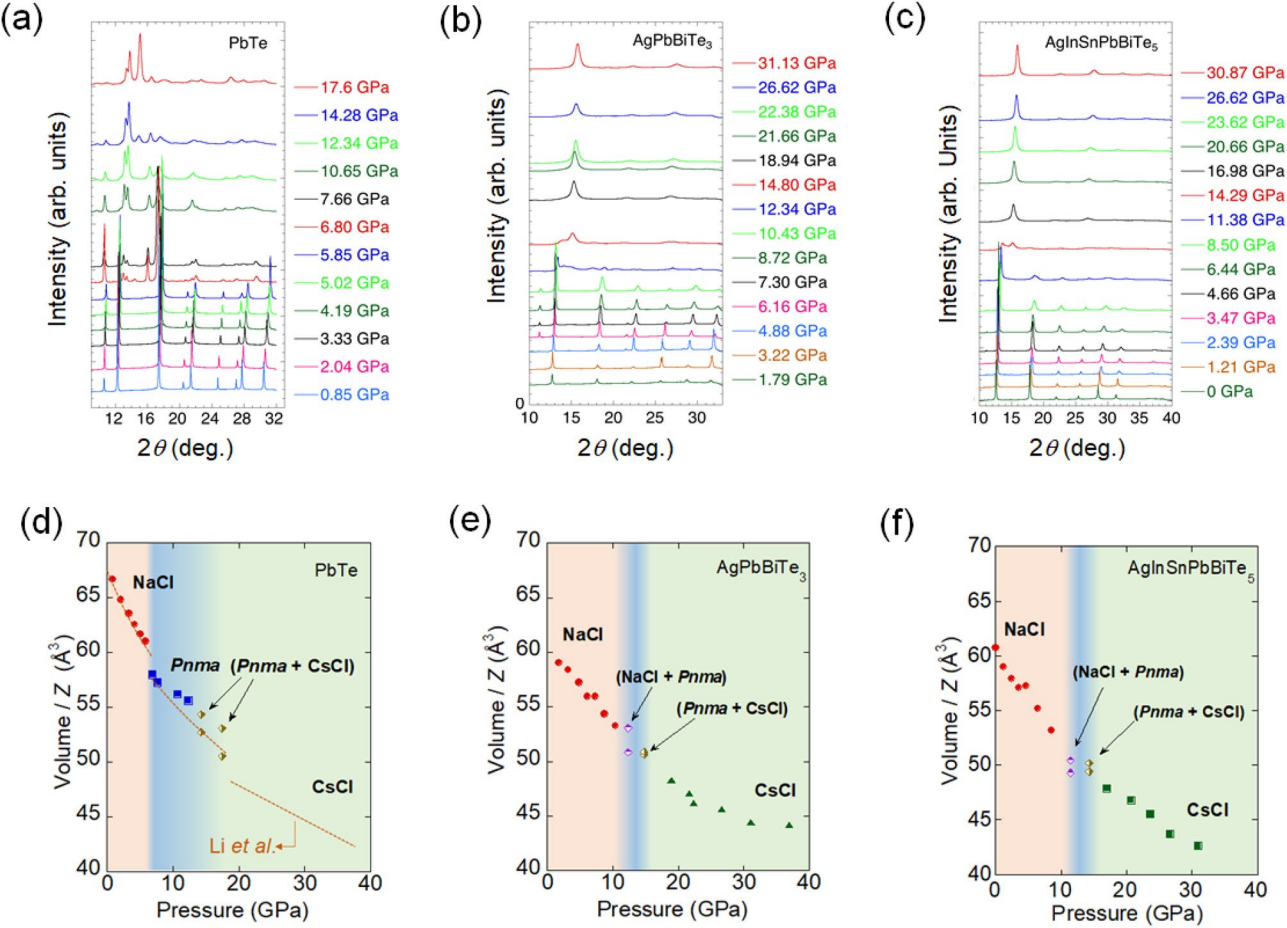
Pressure evolutions of crystal structure for metal tellurides with different configurational entropy of mixing. (**a**–**c**) SXRD patterns for PbTe, AgPbBiTe_3_, and AgInSnPbBiTe_5_. Note that the baseline height of the XRD pattern at each pressure scales to the pressure. (**d–f**) Lattice volumes divided by *Z* (chemical formula sum in a unit cell) for PbTe, AgPbBiTe_3_, and AgInSnPbBiTe_5_ are plotted as a function of pressure. In (**d**), the analysis results reported in Ref.^24^ (Li et al*.*) are represented by orange lines. Structural types are shown in the figures.

Figure 3a–c show the pressure-dependent synchrotron X-ray diffraction (SXRD) patterns for the PbTe, AgPbBiTe_3_, and AgInSnPbBiTe_5_ samples, respectively. For all the SXRD patterns, we performed the Rietveld refinement to confirm the structural type and to evaluate the lattice constant. See Tables S1–S3 and Figs. S4–S6 for details on refinements. On the basis of the refinement results, we established structural phase diagrams under high pressure (Fig. 3d–f) by plotting the pressure dependence of volume per unit formula (*Z*). For PbTe, the structural transition from NaCl-type to *Pnma* occurs at around 6.80 GPa, and the second transition to CsCl-type takes place at 14.28 GPa. Since the transition gradually occurred, the phase diagram contains mixed phases. The results on PbTe are consistent with the previous work by Li et al.^24^. For AgPbBiTe_3_ and AgInSnPbBiTe_5_, similar phase diagrams were obtained, where the NaCl-type structure is stabilized up to ~ 10 GPa, and the *Pnma* phase is suppressed. The pressure where the CsCl-type phase is induced is common to the case of PbTe. In all the structural types including the CsCl-type phase, the lattice volume continuously decreases with pressure. Although the difference in the stability of the NaCl-type and *Pnma* structures may be related to the difference in lattice volume at ambient pressure, we consider that the *Pnma* phase is suppressed, and the NaCl-type phase is stabilized by the effect of alloying at the *M* site. We note that configurational entropy of mixing does not affect the structure of the CsCl-type phase, and lattice volume of the CsCl-type phase commonly decreases with pressure in three *M*Te sample.

### Electronic structure

**Figure 4:**
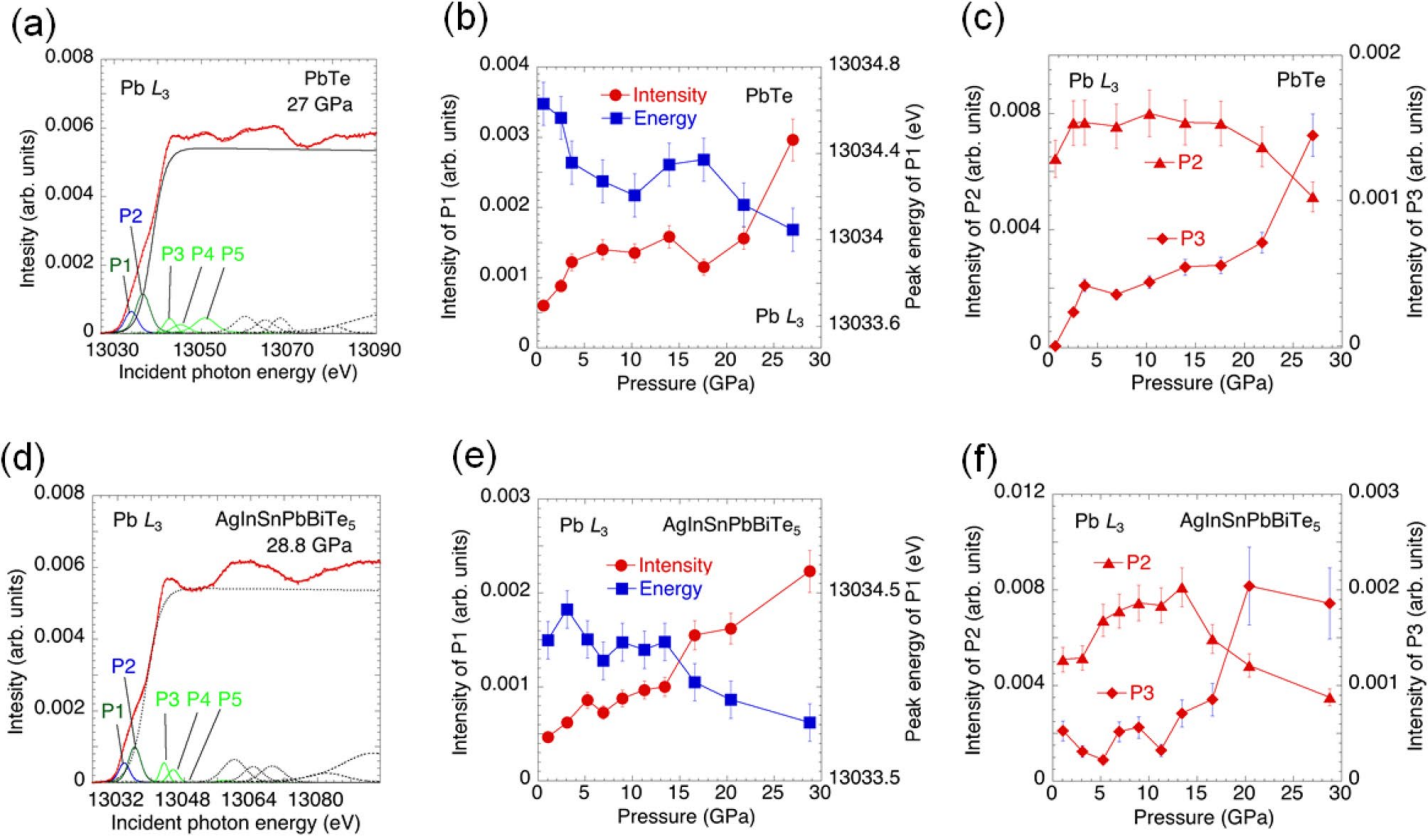
Electronic structure of PbTe and AgInSnPbBiTe_5_ under high pressure. (**a**) Typical Pb-*L*_3_ PFY-XAS spectrum and an example of the fit to the spectrum for PbTe at 27 GPa. (**b**) Pressure dependence of the intensity and the energy of the peak P1 for PbTe. (**c**) Pressure dependence of the intensity of the peaks P2 and P3 for PbTe. (**d**) Typical Pb-*L*_3_ PFY-XAS spectrum and an example of the fit to the spectrum for AgInSnPbBiTe_5_ at 28.8 GPa. (**e**) Pressure dependence of the intensity and the energy of the peak P1 for AgInSnPbBiTe_5_. (**f**) Pressure dependence of the intensity of the peaks P2 and P3 for AgInSnPbBiTe_5_.

To examine the effects of pressure and HEA states on the electronic structure, we performed X-ray absorption spectroscopy with partial fluorescence mode (PFY-XAS) for PbTe and AgInSnPbBiTe_5_. See Fig. S7 for pressure dependences of spectra, and analysis results on the Pb-*L*_3_ and Bi-*L*_3_ spectra. In general, the absorption spectra at the Pb-*L*_3_ absorption edge are similar to those at the Bi-*L*_3_ absorption edge. On the basis of analogically referring to other Pb- or Bi-containing compounds^30,31^, we analyzed the spectra by assuming several peaks. An example of the fit for the PFY-XAS spectra at 27 GPa is shown in Fig. 4a. The PFY-XAS spectra were fitted by assuming some Voigt functions with an arctan-like background^31^. In this study, we focus on the peaks of P1, P2, and P3, where peak P1 could be assigned as a dipole transition of the 2*p*_3/2_ electron into the 6*s* state, and the peaks shown as P2 and P3 correspond to 6*d* states of *t*_2g_ and *e*_g_, respectively^32,33^; we measure 2*p*_3/2_ → *nd* (*n* > 6) transitions at the Pb *L*_3_ absorption edge. There is a *p* density of states (*p* DOS) above the Fermi level, however, we mainly observe the dipole-allowed transitions of Pb 2*p*–6*s* and Pb 2*p*–6*d*, and therefore, the observed spectra do not reflect the Pb 6*p* DOS spectroscopically. The absorption spectra reflect the empty DOS above the Fermi level generally, with a core hole in the final state.

For PbTe with a NaCl-type structure, there is a narrow band gap, and the *p* orbitals of Pb and Te near the Fermi energy are hybridized. See Fig. S8 for the calculated DOS for PbTe^34^. The valence band is mainly composed of Te 5*p* orbitals, and also contains the contribution from Pb 6*p* and 6*s*, while the conduction band is mainly composed of Pb 6*p* orbitals, but also contains Te 5*p* contributions. For PbTe with a CsCl-type structure, band gap is totally closed, and the DOS near the Fermi energy is explained by the contributions from Pb 6*p* and 6*s* orbitals, as well as the Te 5*p* orbitals. Therefore, the pressure evolution of the 6*s* states which could be resolved in our high-resolution spectroscopy, may play an important role on the closing of the band gap as well as the emergence of superconductivity.

The pressure dependences of the intensity and the energy of peak P1 in PbTe is shown in Fig. 4b. The intensity of peak P1 in Fig. 4b gradually increases with pressure, up to about 5 GPa. The increase in the intensity of P1 indicates an increase in the amount of holes in the Pb 6* s* states, which indicates a modification of the band structure. In the middle-pressure phase, (between 5 and 15 GPa) the intensity of P1 does not show a significant change, while it increases remarkably in the HP phase (*P* > 15 GPa). We note that PbTe with a NaCl-type or *Pnma* structure is a semiconductor with a band gap at the low-pressure regime, and we also note that the metallic phase is induced in a CsCl-type structure for pressures above 15 GPa^22,23,34^. The increase of the intensity of P1 corresponds with the increase of the unoccupied 6* s* states of the Pb. This may correlate with the emergence of the superconductivity after the closing of the band gap at *P* > 15 GPa.

On the other hand, the energy of peak P1 shifts to a lower incident energy until it reaches a pressure level of 5 GPa. The incident energy and the intensity do not change in the middle-pressure range of 5–17 GPa, and they start decreasing again for pressures above 18 GPa as shown in Fig. 4b. The shift of the energy of peak P1 to a lower incident energy is explained by the upward shift of the Fermi level or change in the DOS at the Fermi level. Theory suggests that the energy shift of peak P1 may be influenced by the reduction of the band gap^23^ and the theoretical band gap is in the same order as the energy shift of P1 at 5 GPa.

The intensity of P3 (Pb 6*d* DOS, *e*_g_) shows a trend which is similar to the intensity of P1. The intensity of P2 (Pb 6*d* DOS, *t*_2g_) on the other hand decreases with increasing pressure at *P* > 17 GPa (Fig. 4c). It is interesting that there is a large change in the electronic structure for pressures above 20 GPa, but the crystal structure still retains its CsCl-type structure in this pressure range. In PbTe, superconductivity suddenly appears above 18 GPa, and *T*_c_ decreases with pressure monotonically^22^. The present result possibly suggests that the change in the electronic structure is not favorable for the superconductivity of *M*Te, when it has transitioned to the CsCl-type structure.

We also measured the PFY-XAS spectra at the Pb-*L*_3_ absorption edges for AgInSnPbBiTe_5_ as shown in Fig. S7. We observed similar trends in the pressure dependence of the electronic structures as those observed for PbTe. An example of the fit at 28.8 GPa is shown in Fig. 4d. The analysis results on P1, P2, and P3 are plotted in Fig. 4e and f. Figure 4e shows a gradual increase of the P1 intensity with pressure, which is similar to the case of PbTe. The trend of P2 is also similar for the entire pressure range, and that of P3 is basically similar between PbTe and AgInSnPbBiTe_5_. On the other hand, in AgInSnPbBiTe_5_, *T*_c_ increases rapidly in the pressure range of 6–12 GPa and does not show a significant change with further increasing pressure. The pressure-induced change in the *s* DOS (P1 intensity) does not reflect the rapid increase of *T*_c_ at 6–12 GPa as shown in Fig. 4e. The decrease of the P1 energy at 3.1–6.9 GPa may be related to the rapid increase in *T*_c_. The P1 energy also decreases above 13.4 GPa. This suggests the close of the band gap as discussed above for PbTe. The PFY-XAS spectra at the Bi-*L*3 absorption edge were also taken, and the analysis results are summarized in Fig. S7. In AgInSnPbBiTe_5_ the pressure-induced change in the electronic structure seems to be common for Bi and Pb sites; the detailed results are shown under the Supporting Information section. In conclusion, the electronic structures of PbTe and AgInSnPbBiTe_5_ show a similar pressure dependence, even though the structure of PbTe does not change much in the middle-pressure range (*Pnma* + CsCl phase), which disappears in AgInSnPbBiTe_5_. Therefore, the difference in the robustness of superconductivity to pressure in the CsCl-type phase between PbTe and AgInSnPbBiTe_5_ cannot be explained by the pressure evolutions of crystal and electronic structures.

## Discussion

From the structural viewpoint, the impact of the introduction of an HEA site is the suppression of the middle-pressure phase with a *Pnma* structure. In other words, the low-pressure phase with a NaCl-type structure is stabilized up to a higher pressure of *P* > 10 GPa in AgInSnPbBiTe_5_, whereas the NaCl-type phase disappears at ~ 5 GPa for PbTe. Interestingly, the trend of lattice constant in the CsCl-type structure under HP is quite similar for PbTe and AgInSnPbBiTe_5_. However, as revealed in Fig. 2b, the pressure dependences of $T_{{\text{c}}}^{{{\text{onset}}}}$ clearly differ since $T_{{\text{c}}}^{{{\text{onset}}}}$ decreases with pressure for PbTe but does not change largely in the CsCl-type phase for AgInSnPbBiTe_5_. Furthermore, from the electronic-structure viewpoint, we cannot find a clear correlation between the robustness of superconductivity to pressure and the changes in electronic structure under high pressure. Although there is a possibility of the difference of the contribution of the 6*p* states, which could not be measured in our spectra, to the conduction band to increase the number of the carriers in AgInSnPbBiTe_5_.

The results show that the pressure phase diagram of the crystal structure is largely modified by the effect of Δ*S*_mix_. In contrast, the electronic structures are not sensitive to the effect of Δ*S*_mix_. As shown in Fig. S9, it is clear that the difference in the pressure dependence of $T_{{\text{c}}}^{{{\text{onset}}}}$ in the CsCl-type phases is independent of lattice constant, which implies the importance of configurational entropy of mixing modifying local structures. To understand the HEA effects on structural and electronic properties for *M*Te under high pressure, further studies using various probes are needed. However, commonality on the robustness of superconductivity to external pressure in the superconducting HEA (TaNb)_0.67_(HfZrTi)_0.33_^14^ and the HEA-type metal telluride AgInSnPbBiTe_5_ would be demonstrating the universal characteristics of superconductivity in HEA-type materials. Thus, the present results propose that the combination of HP and HEA effects will open a new pathway to the development of new disordered superconductors with exotic superconducting states.

## Conclusion

We have studied the crystal and electronic structure and the robustness of superconducting states in a HEA-type metal telluride (*M*Te; *M* = Ag, In, Sn, Pb, Bi) under high pressure using a polycrystalline sample, and the results were compared with the pressure effects for a middle-entropy system (AgPbBiTe_3_) and a reference system of PbTe. PbTe exhibits a structural transition from a NaCl-type to an orthorhombic *Pnma* structure at low pressures, and further transitions to a CsCl-type structure at high pressures. When the superconductivity of the CsCl-type PbTe is observed, it is found that its superconducting transition temperature (*T*_c_) decreases with pressure. In contrast, in HEA-type AgInSnPbBiTe_5_, *T*_c_ is almost independent of pressure, for pressures ranging from 13.0 to 35.1 GPa. In addition, the middle-entropy system, AgPbBiTe_3_, shows a slight decrease in *T*_c_ with pressure in the CsCl-type structure, which is intermediate trend between PbTe and AgInSnPbBiTe_5_. Those results suggest that the robustness of superconductivity to external pressure has been enhanced by the increase in configurational entropy of mixing at the metal (*M*) site in *M*Te. To further clarify the effects of the modification of the configurational entropy of mixing on the superconducting states and the electronic structure of *M*Te, synchrotron X-ray absorption spectroscopy with partial fluorescence mode (PFY-XAS) for three *M*Te polycrystalline samples of PbTe and AgInSnPbBiTe_5_ were performed. Noticeably, the evolutions of electronic structure under high pressure do not largely differ between PbTe and AgInSnPbBiTe_5_; hence, the difference in the robustness of superconductivity in PbTe and AgInSnPbBiTe_5_ under high pressure is not explained by the difference in their electronic structure. According to the results of this work and previous work on a HEA (Ti–Zr–Hf–Nb–Ta), where the robustness of superconductivity was observed up to ~ 200 GPa, we propose that the robustness of superconductivity under high pressure would be a universal feature in HEA-type superconductors.

## Methods

The polycrystalline sample of PbTe was synthesized by the solid-state reaction of Pb (99.9%) and Te (99.999%) at 900 °C. To obtain a pellet for resistance measurements, pelletizing and second annealing were performed. The polycrystalline samples of AgPbBiTe_3_ and AgInSnPbBiTe_5_ were synthesized using an HP synthesis method where the pressure was kept below 3 GPa, and the temperature kept at 500 °C for 30 min as described in Ref.^20^. The precursor powders of AgPbBiTe_3_ and AgInSnPbBiTe_5_ were synthesized by a solid-state reaction of Ag powders (99.9%) and grains of In (99.99%), Sn (99.999%), Pb (99.9%), Bi (99.999%), and Te (99.999%) at 800 °C, with the nominal compositions.

The electrical resistance measurements were performed at ambient pressure using the conventional four-probe method on a GM refrigerator system. Resistance measurements under high pressure were performed on polycrystalline powder on a Physical Property Measurement System (Quantum Design) using an originally designed diamond anvil cell (DAC) with boron-doped diamond electrodes^35–37^. The sample was placed on the boron-doped diamond electrodes in the center of the bottom anvil. The surface of the bottom anvil, except for the sample space and electrical terminal, were covered with the undoped diamond insulating layer. The cubic boron nitride powders with ruby manometer were used as a pressure-transmitting medium. The applied pressure was estimated by the fluorescence from ruby powders^38^ and the Raman spectrum from the culet of top diamond anvil^39^ using an inVia Raman microscope (RENISHAW). The definition of *T*_c_ is described in Fig. S1.

Pressure dependences of the synchrotron X-ray powder diffraction (SXRD) patterns were measured at BL12B2, SPring-8, using a 3-pin plate diamond anvil cell (DAC, Almax easyLab Industries) with a CCD detection system at room temperature (~ 293 K). Culet size of the diamond anvil was 0.4 mm with a stainless-steel gasket. We took an arrangement of both incoming and outgoing x-ray beams passing through the diamonds with incident photon energy of 18 keV. A two-dimensional image of the CCD system was integrated using the FIT2D program^40^. Silicone oil was used as a pressure-transmitting medium, and pressure was monitored using the ruby fluorescence method^41^. The SXRD data was analyzed through Jana 2006 software^42^ using the Rietveld method.

The pressure dependence of the high-resolution X-ray absorption spectra was measured at beamline BL12XU, SPring-8. Membrane-controlled DACs with a 0.3 mm culet and that with a 0.4 mm culet were used for PbTe and AgInSnPbBiTe_5_, respectively, and silicone oil was used as a pressure-transmitting medium. Beryllium gaskets with a 3 mm diameter were pre-indented at the center. The thickness was approximately 67 μm for PbTe and 31 μm for AgInSnPbBiTe_5_, and the diameters of the sample chamber in the gaskets were approximately 110 μm for PbTe and 140 μm for AgInSnPbBiTe_5_. We employed X-ray absorption spectroscopy (XAS) with a partial fluorescence mode (PFY-XAS), which has an advantage of a higher resolution as compared to that of normal XAS^43,44^. We used the Be gasket in plane geometry where both incoming and outgoing x-ray beams passed through the Be gasket. A Johann-type spectrometer equipped with a spherically bent Si(555) analyzer crystal (radius of ~ 1 m), and a Si solid state detector were used to analyze the Bi *L*α_1_ (10.839 keV, 3*d*_5/2_–2*p*_3/2_) emission at the Bi *L*_3_ absorption edge, and Pb *L*α_1_ (10.551 eV, 3*d*_5/2_–2*p*_3/2_) emission at the Pb *L*_3_ absorption edge^45^. The incident beam is focused at 17 μm × 40 μm by the K-B mirror located at the sample position.

## Supplementary Information

Supplementary Information.

## Data Availability

The data that support the findings of this study are available from the corresponding author (Y.M.) upon reasonable request.

## References

[1] Dubi Y, Meir Y, Avishai Y (2007). Nature of the superconductor–insulator transition in disordered superconductors. Nature.

[2] Gastiasoro MN, Andersen BM (2018). Enhancing superconductivity by disorder. Phys. Rev. B.

[3] Zhao K (2019). Disorder-induced multifractal superconductivity in monolayer niobium dichalcogenides. Nat. Phys..

[4] Mizuguchi Y (2019). Material development and physical properties of bis_2_-based layered compounds. J. Phys. Soc. Jpn..

[5] Mizuguchi Y (2018). Evolution of anisotropic displacement parameters and superconductivity with chemical pressure in BiS_2_-Based REO_0.5_F_0.5_BiS_2_ (RE = La, Ce, Pr, and Nd). J. Phys. Soc. Jpn..

[6] Paris E (2018). Suppression of structural instability in LaOBiS_2−_*x*Se*x* by Se substitution. J. Phys. Condens. Matter.

[7] Nagasaka K (2017). Intrinsic phase diagram of superconductivity in the BiCh_2_-based system without in-plane disorder. J. Phys. Soc. Jpn..

[8] Yeh JW (2004). Nanostructured high-entropy alloys with multiple principal elements: Novel alloy design concepts and outcome. Adv. Energy Mater..

[9] Ye YF (2016). High-entropy alloy: Challenges and prospects. Mater. Today.

[10] Koželj P (2014). Discovery of a superconducting high-entropy alloy. Phys. Rev. Lett..

[11] Sun L, Cava RJ (2019). High-entropy alloy superconductors: Status, opportunities, and challenges. Phys. Rev. Mater..

[12] Kitagawa J, Hamamoto S, Ishizu N (2020). Cutting edge of high-entropy alloy superconductors from the perspective of materials research. Metals.

[13] Stolze K (2018). High-entropy alloy superconductors on an α-Mn lattice. J. Mater. Chem. C.

[14] Guo J (2017). Robust zero resistance in a superconducting high-entropy alloy at pressures up to 190 GPa. PNAS.

[15] Guo J (2019). Record-high superconductivity in niobium-titanium alloy. Adv. Mater..

[16] Mizuguchi Y, Yamashita A (2021). Superconductivity in HEA-Type Compounds.

[17] Sogabe R, Goto Y, Mizuguchi Y (2018). Superconductivity in REO_0.5_F_0.5_BiS_2_ with high-entropy-alloy-type blocking layers. Appl. Phys. Express.

[18] Sogabe R (2019). Improvement of superconducting properties by high mixing entropy at blocking layers in BiS_2_-based superconductor REO_0.5_F_0.5_BiS_2_. Solid State Commun..

[19] Shukunami Y (2020). Synthesis of RE123 high-*T*_c_ superconductors with a high-entropy-alloy-type RE site. Physica C.

[20] Mizuguchi Y (2019). Superconductivity in high-entropy-alloy telluride AgInSnPbBiTe_5_. J. Phys. Soc. Jpn..

[21] Kasem MdR (2020). Superconducting properties of high-entropy-alloy tellurides M-Te (M: Ag, In, Cd, Sn, Sb, Pb, Bi) with a NaCl-type structure. Appl. Phys. Express.

[22] Brandt NB (1975). Superconductivity of the compounds PbTe and PbSe under high pressure. JETP Lett..

[23] Xu L, Zheng Y, Zheng JC (2010). Thermoelectric transport properties of PbTe under pressure. Phys. Rev. B.

[24] Li Y (2013). Phase transitions in PbTe under quasi-hydrostatic pressure up to 50 GPa. High Press. Res..

[25] Fujii Y (1984). A new high-pressure phase of PbTe above 16 GPa. Solid State Commun..

[26] Bencherif Y (2011). High-pressure phases of lead chalcogenides. Mater. Chem. Phys..

[27] Sportouch S (1999). Thermoelectric properties of the cubic family of compounds AgPbBiQ_3_ (Q = S, Se, Te) very low thermal conductivity materials. Mater. Res. Soc. Symp. Proc..

[28] Katsuno M (2020). High-pressure synthesis and superconducting properties of NaCl-type In_1−x_Pb_x_Te (x = 0–0.8). Condens. Matter.

[29] Mitobe T (2021). Superconductivity in In-doped AgSnBiTe_3_ with possible band inversion. Sci. Rep..

[30] Swarbrick JC (2009). High energy resolution X-ray absorption spectroscopy of environmentally relevant lead (II) compounds. Inorg. Chem..

[31] Yamaoka H (2020). Electronic structures of Bi_2_Se_3_ and Ag_x_Bi_2_Se_3_ under pressure studied by high-resolution x-ray absorption spectroscopy and density functional theory calculations. Phys. Rev. B.

[32] Rao KJ, Wong J (1984). A XANES investigation of the bonding of divalent lead in solids. J. Chem. Phys..

[33] Retoux R (1990). Valence state for bismuth in the superconducting bismuth cuprates. Phys. Rev. B.

[34] We obtained the electronic band structures of PbTe using CompES-X, NIMS database https://compes-x.nims.go.jp/.

[35] Matsumoto R (2016). Pressure-induced superconductivity in sulfur-doped SnSe single crystal using boron-doped diamond electrode-prefabricated diamond anvil cell. Rev. Sci. Instrum..

[36] Matsumoto R (2018). Diamond anvil cells using boron-doped diamond electrodes covered with undoped diamond insulating layer. Appl. Phys. Express.

[37] Matsumoto R (2018). Pressure-induced superconductivity in sulfur-doped SnSe single crystal using boron-doped diamond electrode-prefabricated diamond anvil cell. J. Phys. Soc. Jpn..

[38] Irifune T (2003). Ultrahard polycrystalline diamond from graphite. Nature.

[39] Piermarini GJ (1975). Calibration of the pressure dependence of the *R*_1_ ruby fluorescence line to 195 kbar. J. Appl. Phys..

[40] Hammersley AP (1996). Two-dimensional detector software: From real detector to idealised image or two-theta scan. High Press. Res..

[41] Mao HK, Bell PM (1976). High-pressure physics, the 1-megabar mark on the ruby *R*1 static pressure scale. Science.

[42] Petricek V, Dusek M, Palatinus L (2014). Crystallographic computing system JANA2006: general features. Z. Kristallogr..

[43] Hämäläinen K (1991). Elimination of the inner-shell lifetime broadening in x-ray-absorption spectroscopy. Phys. Rev. Lett..

[44] Hämäläinen K (1992). Spin-dependent x-ray absorption of MnO and MnF_2_. Phys. Rev. B.

[45] Yamaoka H (2016). Pressure dependence of the electronic structure of 4*f* and 3*d* electron systems studied by X-ray emission spectroscopy. High Press. Res..

