# Supplementary material for: Robustness of superconductivity to external pressure in high-entropy-alloy-type metal telluride AgInSnPbBiTe5
Source: Sci Rep. 2022 May 12;12:7789. doi: 10.1038/s41598-022-11862-w (PMC9098454; doi:10.1038/s41598-022-11862-w)
Supplement: Supplementary file 1 — Supplementary Information. [file 41598_2022_11862_MOESM1_ESM.pdf]

Supplementary material

Robustness of superconductivity to external pressure in high-entropy-alloy-type metal telluride $AgInSnPbBiTe_5$

Md. Riad Kasem[1], Yuki Nakahira[1], Hitoshi Yamaoka[2], Ryo Matsumoto[3,4], Aichi Yamashita[1], Hirofumi Ishii[5], Nozomu Hiraoka[5], Yoshihiko Takano[4], Yosuke Goto[1], Yoshikazu Mizuguchi[1]*

1. Department of Physics, Tokyo Metropolitan University, 1-1 Minami-Osawa, Hachioji 192-0397, Japan.
2. RIKEN SPring-8 Center, Sayo, Hyogo 679-5148, Japan
3. International Center for Young Scientists (ICYS), National Institute for Materials Science, Tsukuba, Ibaraki 305-0047, Japan
4. International Center for Materials Nanoarchitectonics (MANA), National Institute for Materials Science, Tsukuba, Ibaraki 305-0047, Japan
5. National Synchrotron Radiation Research Center, Hsinchu 30076, Taiwan

* Corresponding author: Y. Mizuguchi

Email: mizugu@tmu.ac.jp

Table S1. Summary of the major and minor phases and the lattice constants revealed by the refinements for PbTe.

PbTe								
P (GPa)	Major phase	Minor phase	Major phase			Minor phase		
			a (Å)	*b* (Å)	*c* (Å)	*a* (Å)	*b* (Å)	*c* (Å)
0.85	NaCl	-	6.41241(7)					
2.04	NaCl	-	6.37689(3)					
3.33	NaCl	-	6.33345(3)					
4.19	NaCl	-	6.30221(6)					
5.02	NaCl	-	6.27128(4)					
5.85	NaCl	*Pnma*	6.24916(5)			8.149(2)	4.5447(11)	6.2867(8)
6.80	*Pnma*	-	8.113(3)	4.5391(2)	6.2977(10)			
7.66	*Pnma*	-	8.009(2)	4.5675(2)	6.263(2)			
10.65	*Pnma*	-	7.931(2)	4.5432(4)	6.2379(12)			
12.34	*Pnma*	-	7.9041(11)	4.5269(5)	6.2161(7)			
14.28	*Pnma*	CsCl	7.849(9)	4.4896(6)	6.1670(4)	3.7498(8)		
17.60	CsCl	*Pnma*	3.69712(10)			7.8775(13)	4.4080(7)	6.1121(3)

Table S2. Summary of the major and minor phases and the lattice constants revealed by the refinements for $AgPbBiTe_3$.

$AgPbBiTe_3$								
P (GPa)	Major phase	Minor phase	Major phase			Minor phase		
			a (Å)	*b* (Å)	*c* (Å)	*a* (Å)	*b* (Å)	*c* (Å)
1.79	NaCl	-	6.18099(10)					
3.22	NaCl	-	6.15772(9)					
4.88	NaCl	-	6.11365(10)					
6.16	NaCl	-	6.07139 (10)					
7.30	NaCl	-	6.07131(11)					
8.72	NaCl	-	6.01276(9)					
10.43	NaCl	-	5.97220(13)					
12.34	NaCl	*Pnma*	5.8814(2)			8.985(2)	3.6292(2)	6.493(2)
14.80	*Pnma*	CsCl	7.5294(9)	4.4553(6)	6.0653(9)	3.6995(4)		
18.94	CsCl	-	3.6377(2)					
21.66	CsCl	-	3.6119(2)					
22.38	CsCl	-	3.58387 (13)					
26.62	CsCl	-	3.5737(2)					
31.13	CsCl	-	3.53490(10)					
36.92	CsCl	-	3.53489(11)					

Table S3. Summary of the major and minor phases and the lattice constants revealed by the refinements for $AgInSnPbBiTe_5$.

$AgInSnPbBiTe_5$								
P (GPa)	Major phase	Minor phase	Major phase			Minor phase		
			a (Å)	*b* (Å)	*c* (Å)	*a* (Å)	*b* (Å)	*c* (Å)
0.00	NaCl	-	6.2386(2)					
1.21	NaCl	-	6.18054(7)					
2.39	NaCl	-	6.14222(7)					
3.47	NaCl	-	6.11298(13)					
4.66	NaCl	-	6.0782(2)					
6.44	NaCl	-	6.0433(2)					
8.50	NaCl	-	5.9682(3)					
11.38	NaCl	*Pnma*	5.86438(7)			7.918(2)	4.2683(5)	5.836(2)
14.29	*Pnma*	CsCl	9.2478 (11)	3.6954(3)	5.8745 (4)	3.6694 (2)		
16.98	CsCl	-	3.6309 (2)					
20.66	CsCl	-	3.60484(13)					
23.62	CsCl	-	3.57124(9)					
26.62	CsCl	-	3.52154(11)					
30.87	CsCl	-	3.49282(8)					

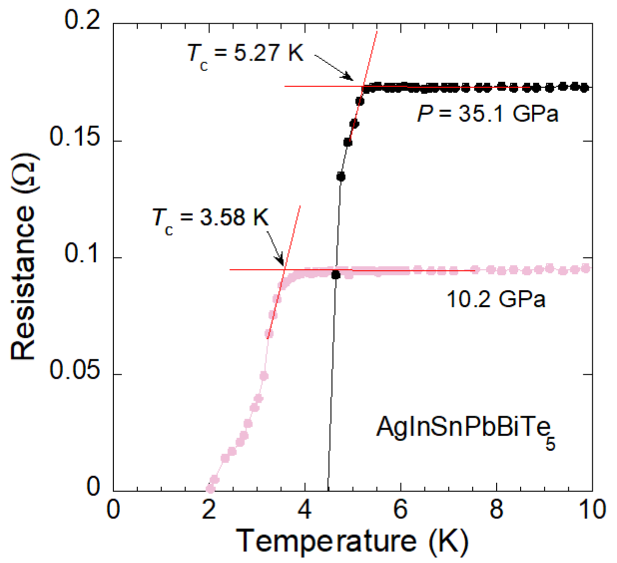

Fig. S1. Estimation of T_c from the DAC resistance data of $AgInSnPbBiTe_5$. T_c was estimated form a cross point of two (red) lines.

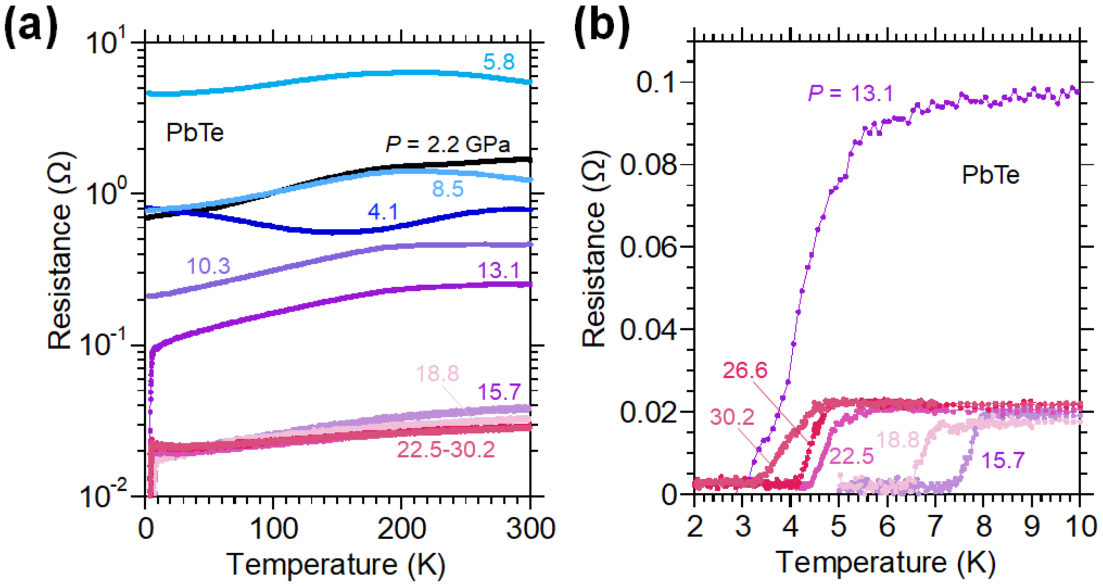

Fig. S2. Temperature dependences of electrical resistance for PbTe under high pressures.

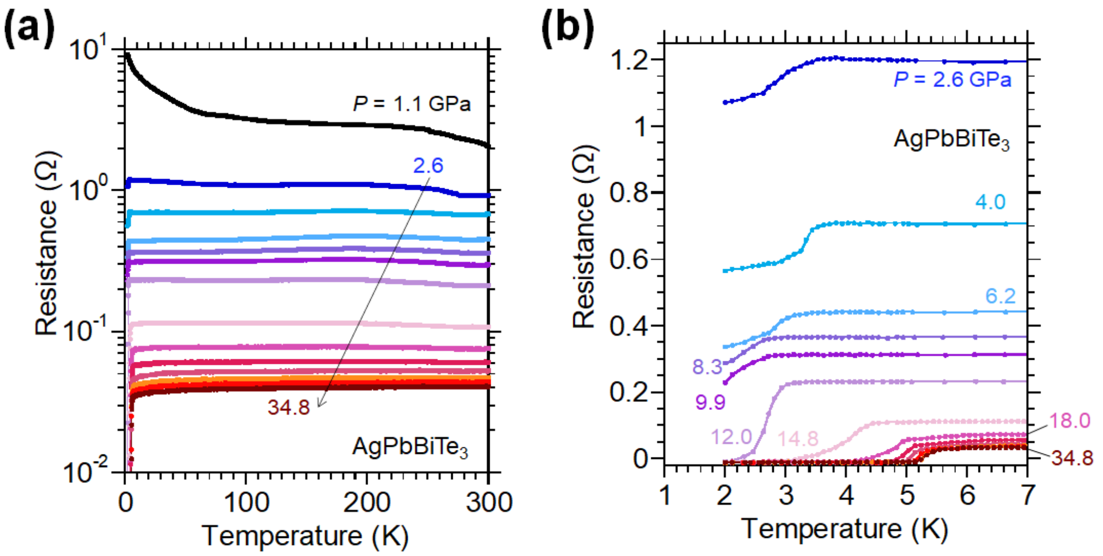

Fig. S3. Temperature dependences of electrical resistance for $AgPbBiTe_3$ under high pressures.

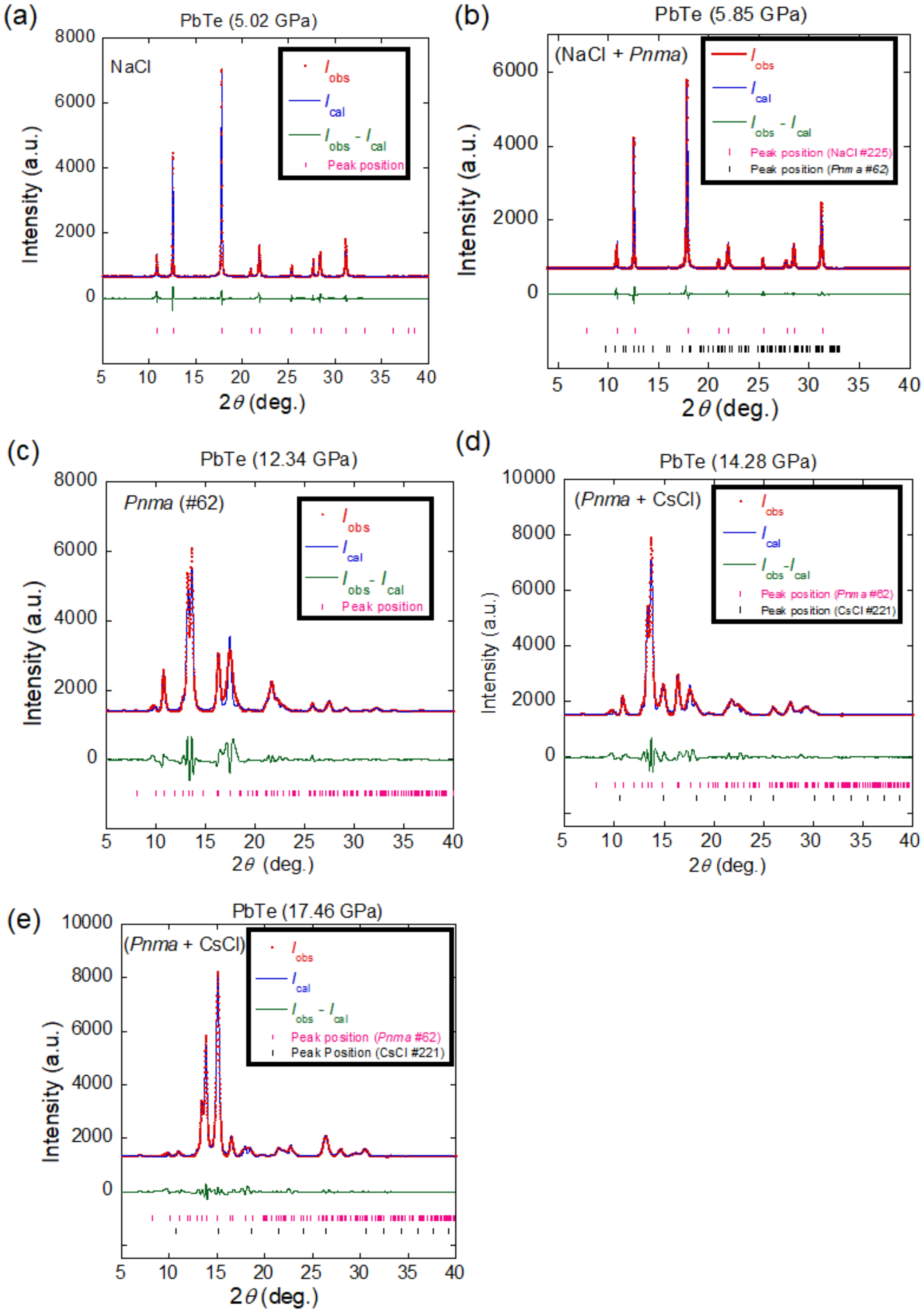

Fig. S4. Typical refinement results for PbTe.

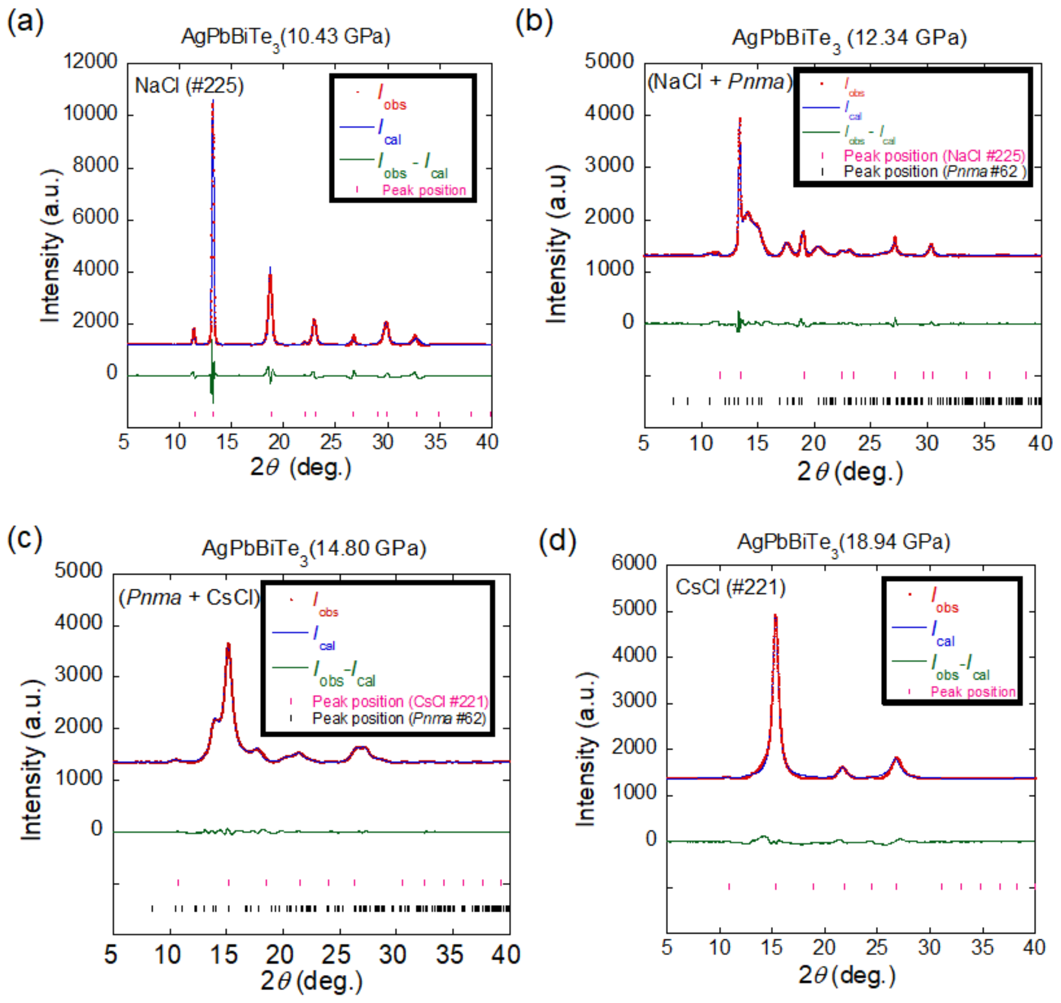

Fig. S5. Typical refinement results for $AgPbBiTe_3$.

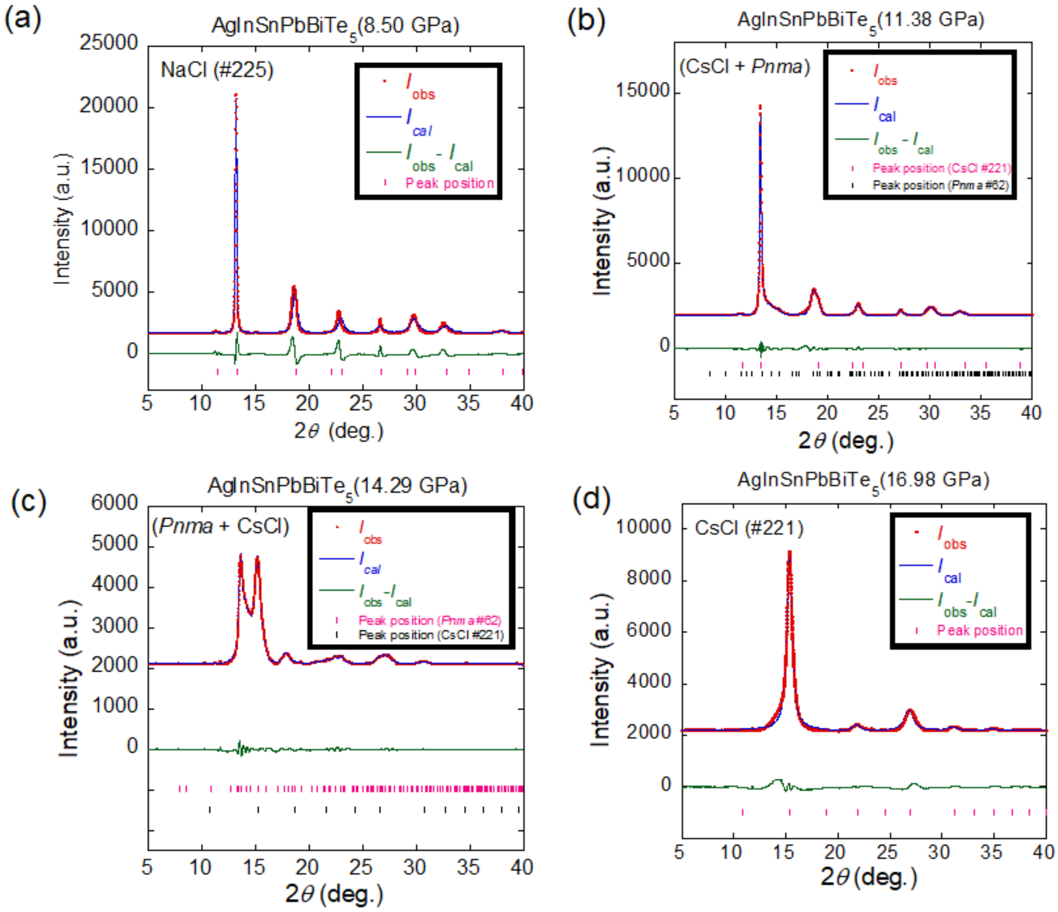

Fig. S6. Typical refinement results for $AgPbBiTe_3$.

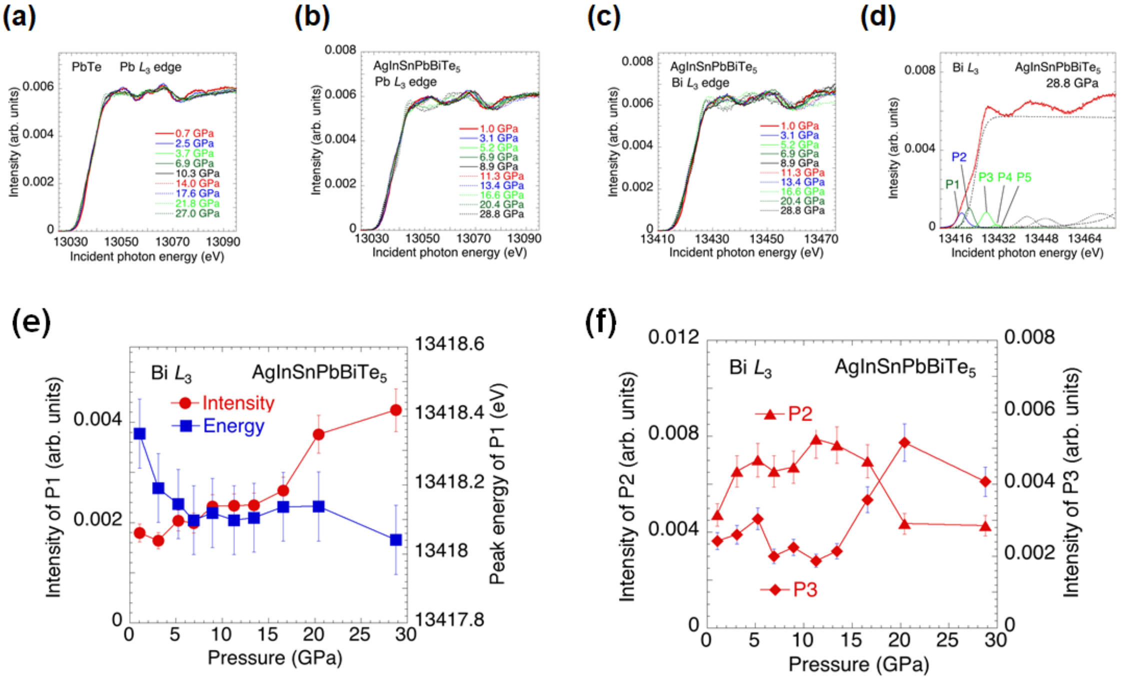

Fig. S7. (a,b) Pressure dependence of the PFY-XAS spectra at the Pb-L_3 absorption edge for PbTe, and $AgInSnPbBiTe_5$. (c) Pressure dependence of the PFY-XAS spectra at the Bi-L_3 absorption edge for $AgInSnPbBiTe_5$. (d) An example of the Bi-L_3 PFY-XAS spectrum at 28.8 GPa. (e,f) Pressure dependence of the intensity and the energy for the peaks P1, P2, and P3 obtained from the Bi-L_3 PFY-XAS data for $AgInSnPbBiTe_5$.

As shown in Fig. S7, the intensity of P1 increases with pressure in the whole pressure range measured in this study. Both intensities of P2 and P3 show a trend to increase in the NaCl-type structure phase up to ~15 GPa. The intensity of P2 decreases with pressure with pressure above ~15 GPa in the in the CsCl-type structure phase, while that of P3 decreases. The energy of P1 show a trend to decrease with pressure in both Bi and Pb sites, although that of P1 of Bi does not change at the pressure range between 7-20 GPa.

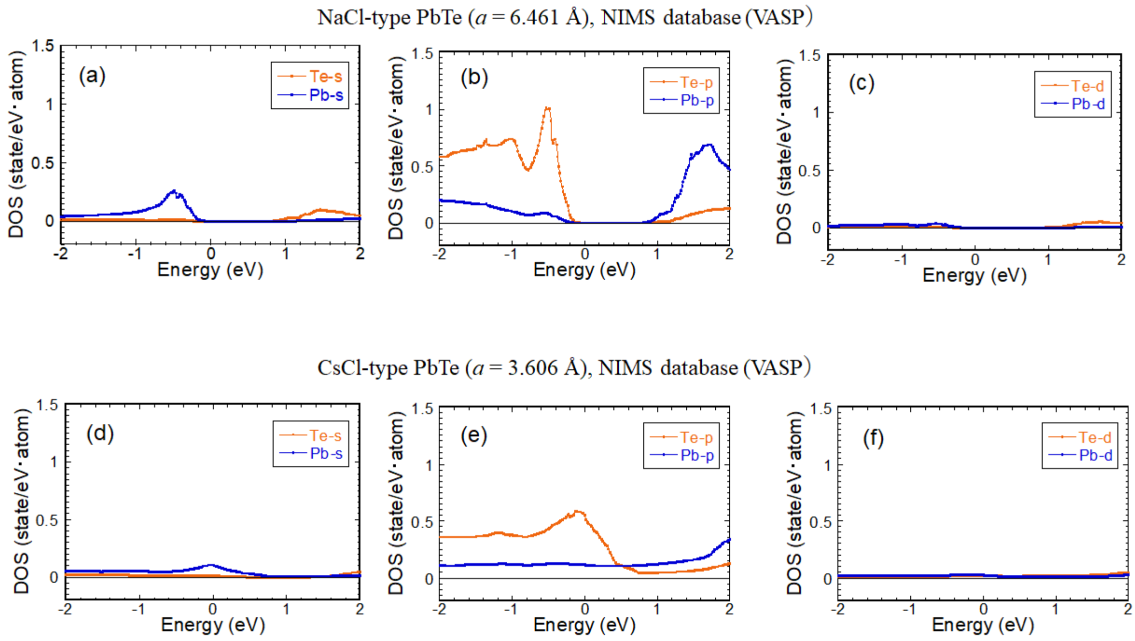

Fig. S8. (a–c) Calculated s, p, d density of states (DOS) of PbTe with a NaCl-type structure. (d–f) Calculated s, p, d density of states (DOS) of PbTe with a CsCl-type structure. The electronic density of states was calculated by CompES-X, NIMS database (https://compes-x.nims.go.jp/index.html) on 29 September 2021.

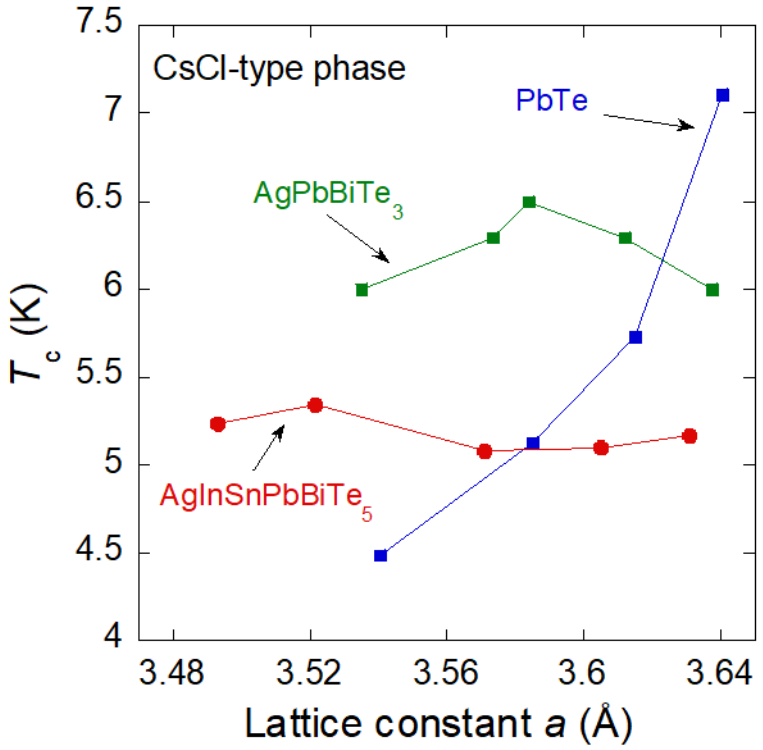

Fig. S9. Lattice constant dependence of T_c^{onset} in the CsCl-type phases of PbTe, $AgPbBiTe_3$, and $AgInSnPbBiTe_5$.
